# Integrated Application of Cattle Manure or Microbial Inoculants with Chemical Fertilizers Improves Nutrient Cycling in Albic Soils and Enhances Nutrient Use Efficiency and Yield in a Maize–Soybean Rotation System

**DOI:** 10.3390/plants15050684

**Published:** 2026-02-25

**Authors:** Hao Li, Qu Chen, Yuzhe Wu, Yubo Sun, Da Song, Lining Dou, Meng Hou, Shoukun Song, Jingru Zheng, Yuxian Zhang, Mingcong Zhang, Tangzhe Nie, Xingchao Liu, Mengxue Wang

**Affiliations:** 1 College of Agronomy, Heilongjiang Bayi Agricultural University, Daqing 163000, China; 17604530392@163.com (H.L.); 18545559306@163.com (Y.W.); 15776586250@163.com (Y.S.); songda200205@163.com (D.S.); 13836216629@163.com (L.D.); houmeng@byau.edu.cn (M.H.); a13199436890@163.com (S.S.); lvsebaowen513@163.com (J.Z.); zyx_lxy@126.com (Y.Z.); zhangmingcong@163.com (M.Z.); 2 Engineering Research Center of Agricultural Microbiology Technology, Ministry of Education & Heilongjiang Provincial Key Laboratory of Ecological Restoration and Resource Utilization for Cold Region & School of Life Sciences, Heilongjiang University, Harbin 150080, China; 18345743002@163.com; 3 National Multigrain Engineering and Technology Center, Daqing 163000, China; 4 School of Water Conservancy and Electric Power, Heilongjiang University, Harbin 150080, China; 2019036@hlju.edu.cn; 5 School of Water Conservancy and Civil Engineering, Northesat Agricultural University, Harbin 150030, China; dnlxc@neau.edu.cn

**Keywords:** nutrient use efficiency, microbial fertilization, soil nutrient dynamics, Albic soil, Sanjiang Plain

## Abstract

Soil quality degradation and low nutrient use efficiency constrain sustainable maize–soybean rotation in the Albic soil region of Northeast China. A field experiment was conducted in 2023–2024 at Qixing Farm (Jiansanjiang, Heilongjiang, China) to evaluate chemical fertilizer combined with cattle manure or microbial inoculants. Five treatments were established: no fertilization (CK), chemical fertilizer alone (CF), chemical fertilizer combined with cattle manure (CF+CM), chemical fertilizer combined with a *Bacillus subtilis* inoculant (CF+CRA), and chemical fertilizer combined with a *Bacillus megaterium* inoculant (CF+CRB). Soil available nutrient dynamics, crop nutrient accumulation and translocation, fertilizer use efficiency, and yield were assessed. In maize, CF+CRB significantly enhanced pre-anthesis N translocation and post-anthesis P accumulation, increasing grain yield to 14,533 kg ha^−1^ (+28.6% vs. CF). In soybean, CF+CRB produced 3328.15 kg ha^−1^, 15.8% higher than CF. CF+CRA significantly increased soil available P during the soybean flowering-pod stage and improved K allocation at later stages. Overall, integrating chemical fertilizer with CRB improved yield and nutrient use efficiency. Based on crop-specific nutrient requirements, CRB is recommended for the maize season to strengthen nutrient translocation, whereas cattle manure or CRA can be applied in the soybean season to sustain K supply.

## 1. Introduction

The maize–soybean rotation system is an important agricultural model for securing global supplies of both food and edible oil. Northeast China is one of the world’s major grain-producing regions, and its stable regional production capacity is essential for global food security [1,2]. Heilongjiang Province, located in the Sanjiang Plain, is a major grain-producing region with widespread distribution of Albic soils. This soil type is typically of low to medium fertility, characterized by high acidity, high clay content, relatively compact structure, and poor aeration [3,4]. These properties lead to unstable water and nutrient availability, reduce fertilizer use efficiency, and constrain crop productivity [5,6]. Additionally, long-term intensive cultivation has heavily relied on chemical fertilizers, resulting in low nitrogen and phosphorus use efficiency, soil degradation, and disrupted nutrient cycling [7,8,9]. These problems not only weaken the sustainability of the rotation system but also hinder the adoption of more environmentally sustainable agricultural practices. Therefore, improving nutrient cycling and fertilizer use efficiency while maintaining rotational productivity is a key scientific and practical challenge for promoting resource-efficient and environmentally sustainable agricultural production in typical Albic soil regions of Northeast China.

Within rotation systems, the stability of crop yields is not only influenced by nutrient input levels but also by nutrient supply, absorption, and allocation processes within the soil and crop system [10,11,12,13]. In the Albic soil region, compact structure and poor aeration may restrict soil–crop nutrient cycling, causing pronounced temporal and spatial fluctuations in plant-available nutrients. Such fluctuations can exacerbate mismatches between nutrient supply and crop demand during critical growth stages, thereby further reducing fertilizer use efficiency [14,15].

Maize and soybean differ markedly in growth patterns and nutrient requirements [16,17]. In maize, grain formation depends on nutrient accumulation and remobilization from pre-flowering organs, together with continued nutrient uptake after flowering to support grain filling [18,19]. As a leguminous crop, soybean’s yield is highly sensitive to nutrient supply during key reproductive growth stages [20,21]. When soil nutrient availability fails to meet crop demand at critical stages, or when within-plant nutrient transport and allocation are inefficient, fertilizer use efficiency may decline and yield potential may be limited [22].

Organic materials and microbial inoculants are widely considered promising approaches to improve soil nutrient availability, enhance the crop growth environment, and increase resource-use efficiency [23,24]. As an organic fertilizer, cattle manure supplies N, P, and K and also provides mineralizable organic N and labile carbon. These inputs can stimulate N transformation processes and influence overall N cycling. As a result, cattle manure may regulate the temporal dynamics of mineral nitrogen supply and provide a more sustained nutrient supply during later growth stages [25]. Under long-term or combined application scenarios, cattle manure can promote the accumulation of soil organic matter, improve nutrient retention, and reduce the risk of nutrient loss (such as nitrogen loss), thereby enhancing nutrient cycling efficiency at the system level [26]. *Bacillus* species are widely used in nutrient management due to their adaptability and rhizosphere-colonization capacity [27]. Functional bacteria such as *Bacillus megaterium*, *Bacillus subtilis*, and *Bacillus amyloliquefaciens* participate in nutrient cycling by activating, transforming, and promoting nutrient absorption. *Bacillus megaterium* can secrete organic acids, phosphatases, and phytases to activate insoluble phosphorus [28]. *Bacillus subtilis*, a typical plant growth-promoting rhizobacterium, can enhance soil enzyme activities and microbial activity, promote nitrogen turnover, and improve rhizosphere nitrogen supply [29,30]. *Bacillus amyloliquefaciens* can enhance the availability of soil nutrients, facilitate the release and uptake of nitrogen, phosphorus and other elements [31]. Therefore, in crop rotation systems, chemical fertilizers provide a source of available nutrients, while cattle manure and *Bacillus* inoculants may regulate the supply dynamics of available N, P, and K in the soil, as well as nutrient accumulation and transport in crops.

Although many studies have investigated the effects of organic fertilizers or microbial inoculants on single-crop production and soil improvement [32,33,34,35], there is still a lack of research on the effects of combined chemical fertilizers with cattle manure or microbial inoculants on soil available nutrient dynamics, crop nutrient accumulation, and nutrient transport characteristics before and after flowering, which ultimately determine fertilizer use efficiency and yield formation in maize–soybean rotation systems in the typical Albic soil region of the Sanjiang Plain in Heilongjiang Province, China. In particular, integrated studies linking seasonal soil nutrient supply dynamics, within-plant nutrient redistribution, and yield remain scarce. Based on this background, a maize–soybean rotation field experiment was conducted from 2023 to 2024 at Qixing Farm in the Jiansanjiang Reclamation Area of the Sanjiang Plain, Heilongjiang Province, China. The experiment included five treatments: no fertilization (CK), chemical fertilizer alone (CF), chemical fertilizer plus cattle manure (CF+CM), and chemical fertilizer combined with two microbial inoculants (CF+CRA and CF+CRB). During the crop growth period, we monitored the temporal dynamics of soil alkali-hydrolyzable N, available P, and available K, and we quantified aboveground accumulation of nutrients (e.g., N and P) and characterized nutrient translocation before and after flowering. We evaluated N and P fertilizer use efficiency and the yield responses of maize and soybean, and compared differences among the combined treatments in nutrient utilization as well as yield. The objective of this study was to elucidate, from the perspective of fertilizer use efficiency and nutrient balance, how different fertilization regimes affect soil available nutrient status in Albic soils, fertilizer use efficiency, nutrient accumulation and translocation, and crop yield. The findings are expected to provide a theoretical basis and practical reference for reduced-input, higher-efficiency management and green agricultural development of maize–soybean rotation systems in the Albic soil region of Northeast China.

## 2. Results

### 2.1. Effects of Chemical Fertilizer Combined with Cattle Manure or Microbial Conditioners on Soil Available Nutrients in a Maize–Soybean Rotation System

**Figure 1:**
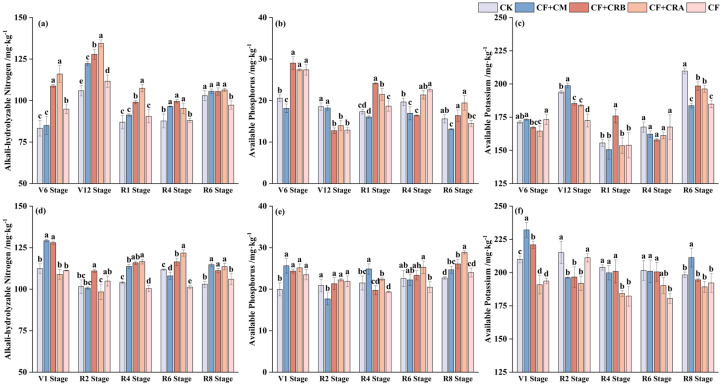
Effects of Combined Application of Cattle Manure and Microbial Inoculants with Chemical Fertilizers on Soil Available Nutrient Contents in a Maize–Soybean Rotation System: (**a**) Soil alkali-hydrolyzable N in 2023; (**b**) Soil alkali-hydrolyzable N in 2024; (**c**) Soil available P in 2023; (**d**) Soil available P in 2024; (**e**) Soil available K in 2023; (**f**) Soil available K in 2024. Treatments: CK (control); CF (chemical fertilizer); CF+CM (CF + cattle manure); CF+CRA (CF + Complex Conditioner A); CF+CRB (CF + Complex Conditioner B). Values are presented as the mean ± standard deviation (SD) of three replicates (*n* = 3). Error bars indicate SD. Different lowercase letters above bars within the same panel indicate significant differences among treatments (*p* < 0.05; Waller–Duncan multiple comparison test following one-way ANOVA).

As shown in Figure 1, soil available nutrients exhibited pronounced seasonal fluctuations across the two growing seasons. Fertilization treatments altered both the timing and magnitude of peak values.

In the maize season, alkali-hydrolyzable N peaked at V6 under Complex Conditioner B (CF+CRB) and Complex Conditioner A (CF+CRA), which were significantly higher than CF by 14.7% and 22.3%, respectively. At V12, CF+CRA reached its maximum and remained significantly higher than CF by 20.4%; CF+CRB and CF+CM were also significantly higher than CF. In the soybean season, CF+CRA showed the highest alkali-hydrolyzable N at R6, exceeding CF by 20.3%. At R8, CF+CM had the highest value, 8.3% higher than CF.

In the 2023 maize season, available P was highest at V12 under CK and CF+CM, whereas CF+CRA was significantly lower than CK by 24.6%. At R4, CF showed the highest available P and was significantly higher than CF+CM by 33.7%. In the 2024 soybean season, CF+CRA significantly increased available P at R2 by 45.1% relative to CK, and it remained the highest at R6 and R8. In contrast, CF+CM showed the lowest available P at R8.

In the 2023 maize season, available K was highest at V12 under CF+CM, which was 2.5% higher than CK, while CF+CRA was 5.2% lower than CK. At R6, CK had the highest available K, whereas CF+CM had the lowest. In the 2024 soybean season, CK and CF were significantly higher than the other treatments at R2. At R8, CF+CM had the highest available K, 6.5% higher than CK, while CF+CRA was 4.5% lower than CK.

Overall, treatments combined with organic amendments showed nutrient- and stage-specific advantages. CF+CRA consistently improved alkali-hydrolyzable N and available P during the mid-to-late growth stages, whereas CF+CM performed better in maintaining soil available K levels.

### 2.2. Effects of Chemical Fertilizer Combined with Cattle Manure or Microbial Conditioners on Aboveground Dry Matter Accumulation of Maize and Soybean in a Maize–Soybean Rotation System

**Figure 2:**
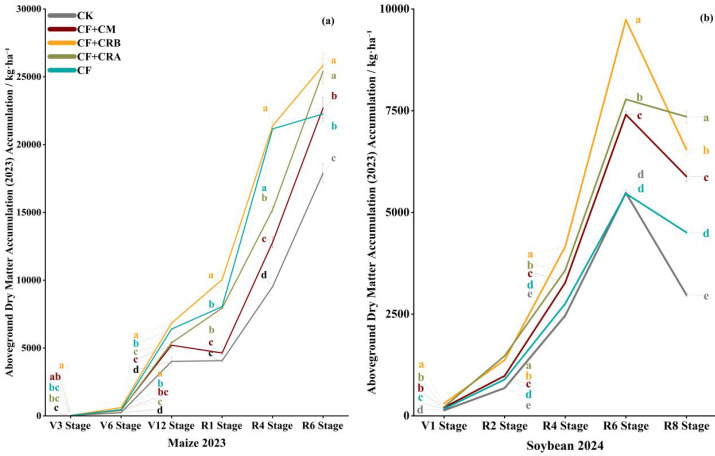
Effects of Combined Application of Cattle Manure and Microbial Amendments with Chemical Fertilizers on Aboveground Dry Matter Accumulation of Maize and Soybean in a Maize–Soybean Rotation System: (**a**) Aboveground dry matter accumulation of maize during the 2023 growing season; (**b**) Aboveground dry matter accumulation of soybean during the 2024 growing season. Treatments: CK (control); CF (chemical fertilizer); CF+CM (CF + cattle manure); CF+CRA (CF + Complex Conditioner A); CF+CRB (CF + Complex Conditioner B). Values are presented as the mean ± standard deviation (SD) of three replicates (*n* = 3). Error bars indicate SD. Different lowercase letters above bars within the same panel indicate significant differences among treatments (*p* < 0.05; Waller–Duncan multiple comparison test following one-way ANOVA).

Aboveground dry matter accumulation increased continuously with crop development in both growing seasons, while significant differences among fertilization treatments were observed at key growth stages (Figure 2). In the maize season, the promoting effect of the combined treatments was more evident at V6 and R1. At R6, CF+CRB reached 25,839 kg·ha^−1^, which was significantly higher than CF by 16.1%. In the soybean season, treatment differences were mainly observed during R4–R8. CF+CRB showed the highest accumulation at R6, whereas CF+CRA was highest at R8. At maturity, all three combined treatments were significantly higher than CF. Overall, combining chemical fertilizer with cattle manure or microbial conditioners increased dry matter accumulation at critical stages and at maturity in the rotation system.

Specifically, in the maize season, aboveground dry matter increased progressively over time. At V6, CF+CRB was the highest and exceeded CF by 32.0%. At R1, CF+CRB remained significantly higher than CF by 24.8%. By R6, dry matter under CF+CRB reached 25,839.25 kg·ha^−1^, significantly higher than CF (+16.1%). At R6, CF+CRA did not differ significantly from CF+CRB, whereas CF+CM showed relatively lower accumulation.

In the soybean season, CF+CRB produced the highest dry matter at R4 (4163.39 kg·ha^−1^), significantly higher than CF by 50.8%. At R6, CF+CRB was 78.1% higher than CF. At R8, CF+CRA showed the highest accumulation, exceeding CF by 63.1%, while CF+CRB and CF+CM were also significantly higher than CF. These results indicate that the combined treatments maintained a dry matter accumulation advantage during late reproductive growth in soybean.

### 2.3. Effects of Chemical Fertilizer Combined with Cattle Manure or Microbial Conditioners on Aboveground Nitrogen Accumulation of Maize and Soybean in a Maize–Soybean Rotation System

**Figure 3:**
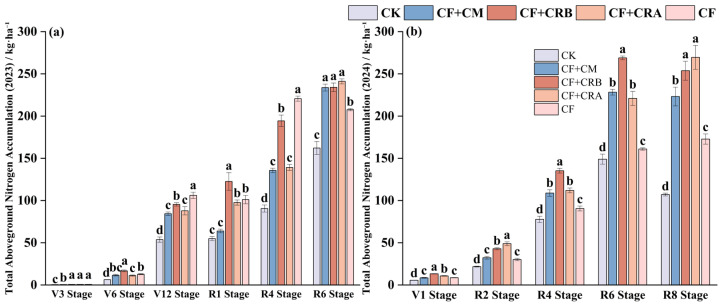
Effects of Combined Application of Cattle Manure and Microbial Amendments with Chemical Fertilizers on Aboveground Total Nitrogen Accumulation in a Maize–Soybean Rotation System: (**a**) Maize in the 2023 season; (**b**) Soybean in the 2024 season. Treatments: CK (control); CF (chemical fertilizer); CF+CM (CF + cattle manure); CF+CRA (CF + Complex Conditioner A); CF+CRB (CF + Complex Conditioner B). Values are presented as the mean ± standard deviation (SD) of three replicates (*n* = 3). Error bars indicate SD. Different lowercase letters above bars within the same panel indicate significant differences among treatments (*p* < 0.05; Waller–Duncan multiple comparison test following one-way ANOVA).

**Figure 4:**
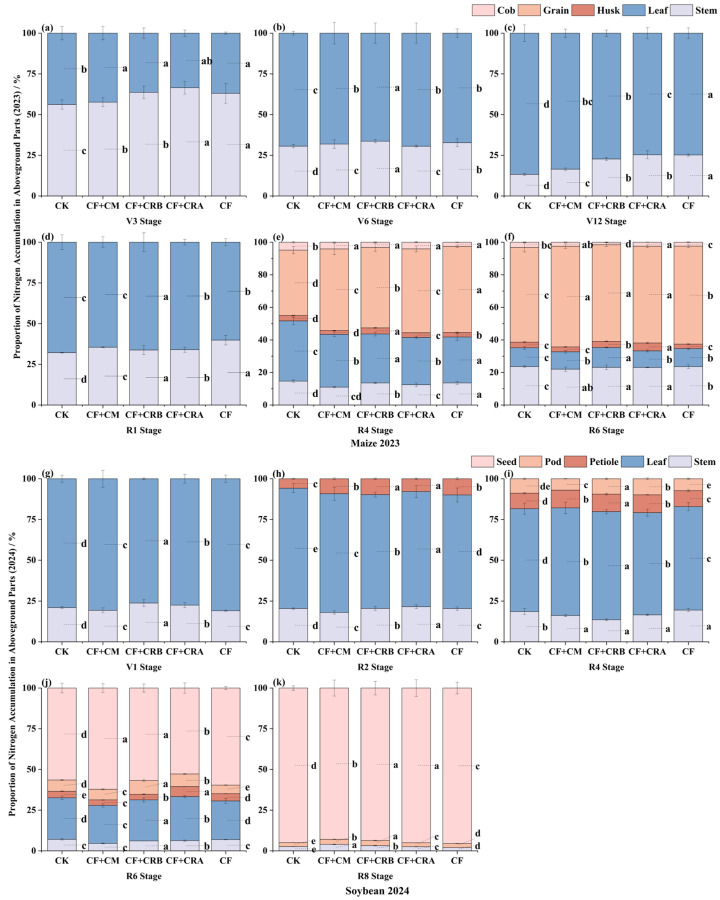
Effects of Combined Application of Cattle Manure and Microbial Amendments with Chemical Fertilizers on the Proportion of Nitrogen Accumulation in Different Aboveground Plant Parts in a Maize–Soybean Rotation System: (**a**) Maize at the V3 stage in 2023; (**b**) Maize at the V6 stage in 2023; (**c**) Maize at the V12 stage in 2023; (**d**) Maize at the R1 stage in 2023; (**e**) Maize at the R4 stage in 2023; (**f**) Maize at the R6 stage in 2023; (**g**) Soybean at the V1 stage in 2024; (**h**) Soybean at the R2 stage in 2024; (**i**) Soybean at the R4 stage in 2024; (**j**) Soybean at the R6 stage in 2024; (**k**) Soybean at the R8 stage in 2024. **Note:** Soybean has no seed tissue at the V1, R2, and R4 stages; therefore, the seed (grain) category is not shown in these panels and only appears from R6 and R8 onward. Treatments: CK (control); CF (chemical fertilizer); CF+CM (CF + cattle manure); CF+CRA (CF + Complex Conditioner A); CF+CRB (CF + Complex Conditioner B). Values are presented as the mean ± standard deviation (SD) of three replicates (*n* = 3). Error bars indicate SD. Different lowercase letters above bars within the same panel indicate significant differences among treatments (*p* < 0.05; Waller–Duncan multiple comparison test following one-way ANOVA).

As shown in Figure 3 and Figure 4, aboveground N accumulation increased with crop development during the maize season, with significant treatment differences at key growth stages. Compared with CF, CF+CRB significantly increased N accumulation by 30.4% at V6 and by 21.2% at R1. At R6, CF+CRB and CF+CRA showed the highest N accumulation, exceeding CF by 12.8% and 16.2%, respectively. Across treatments, N partitioning among plant organs followed a similar pattern: from V6 to V12, N was mainly allocated to leaves, whereas grains became the dominant N sink at maturity.

During the soybean season, the combined treatments significantly enhanced N accumulation at key stages. CF+CRB increased N accumulation relative to CF by 53.1% at V1, 49.4% at R4, and 67.1% at R6. In contrast, CF+CRA achieved the highest N accumulation at R2 and R8, exceeding CF by 62.0% and 55.9%, respectively. Nitrogen partitioning showed the same trend as in maize: leaves accounted for a large proportion at early stages, while the grain proportion increased markedly at maturity, with consistently high grain N allocation across treatments.

Specifically, at V12 in maize, leaves represented the major N pool (e.g., under CF, leaf N accounted for 74.8% of aboveground N). At maturity, grains became the primary N pool (e.g., under CF+CRB, grain N accounted for 59.4%, and grain N accumulation reached 138.97 kg·ha^−1^). In soybean, the grain N proportion increased sharply at maturity and reached 95.1% under CF+CRA.

### 2.4. Effects of Chemical Fertilizer Combined with Cattle Manure or Microbial Conditioners on Aboveground Phosphorus Accumulation of Maize and Soybean in a Maize–Soybean Rotation System

**Figure 5:**
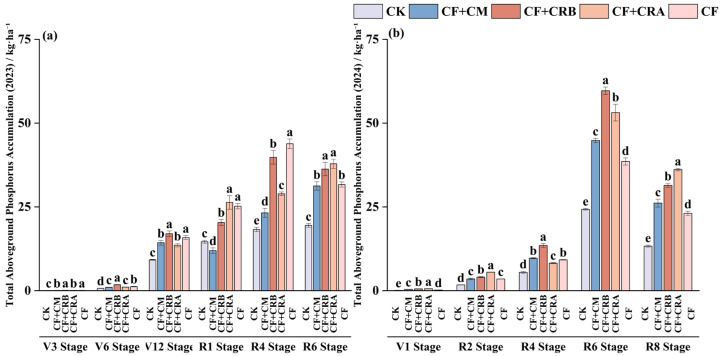
Effects of Combined Application of Cattle Manure and Microbial Amendments with Chemical Fertilizers on Aboveground Total Phosphorus Accumulation in a Maize–Soybean Rotation System: (**a**) Maize in the 2023 season; (**b**) Soybean in the 2024 season. **Note:** Soybean has no seed tissue at the V1, R2, and R4 stages; therefore, the seed (grain) category is not shown in these panels and only appears from R6 and R8 onward. Treatments: CK (control); CF (chemical fertilizer); CF+CM (CF + cattle manure); CF+CRA (CF + Complex Conditioner A); CF+CRB (CF + Complex Conditioner B). Values are presented as the mean ± standard deviation (SD) of three replicates (*n* = 3). Error bars indicate SD. Different lowercase letters above bars within the same panel indicate significant differences among treatments (*p* < 0.05; Waller–Duncan multiple comparison test following one-way ANOVA).

**Figure 6:**
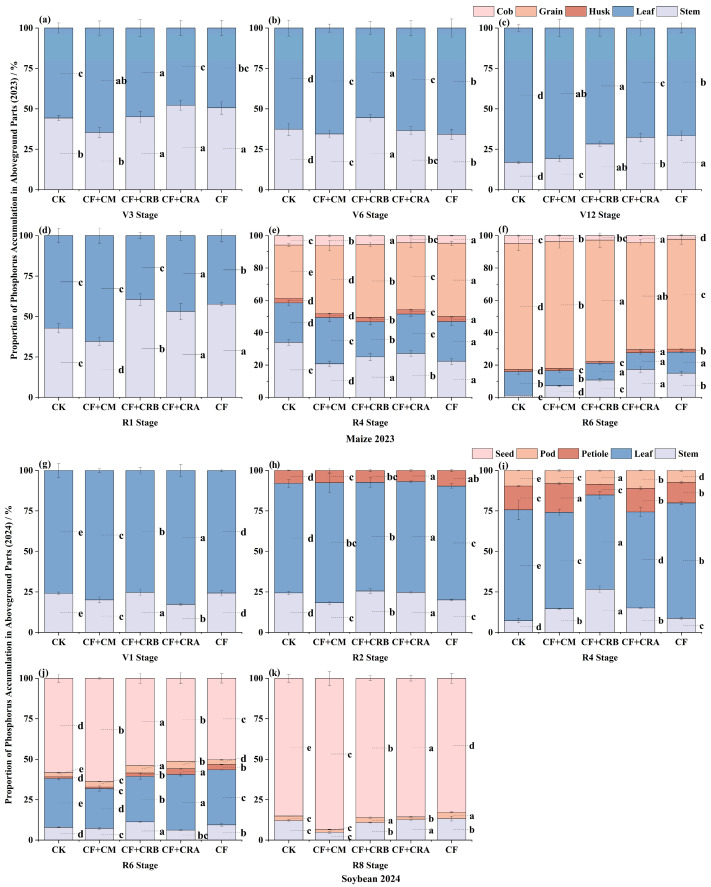
Effects of Combined Application of Cattle Manure and Microbial Amendments with Chemical Fertilizers on the Proportion of Phosphorus Accumulation in Different Aboveground Plant Parts in a Maize–Soybean Rotation System: (**a**) Maize at the V3 stage in 2023; (**b**) Maize at the V6 stage in 2023; (**c**) Maize at the V12 stage in 2023; (**d**) Maize at the R1 stage in 2023; (**e**) Maize at the R4 stage in 2023; (**f**) Maize at the R6 stage in 2023; (**g**) Soybean at the V1 stage in 2024; (**h**) Soybean at the R2 stage in 2024; (**i**) Soybean at the R4 stage in 2024; (**j**) Soybean at the R6 stage in 2024; (**k**) Soybean at the R8 stage in 2024. **Note:** Soybean has no seed tissue at the V1, R2, and R4 stages; therefore, the seed (grain) category is not shown in these panels and only appears from R6 and R8 onward. Treatments: CK (control); CF (chemical fertilizer); CF+CM (CF + cattle manure); CF+CRA (CF + Complex Conditioner A); CF+CRB (CF + Complex Conditioner B). Values are presented as the mean ± standard deviation (SD) of three replicates (*n* = 3). Error bars indicate SD. Different lowercase letters above bars within the same panel indicate significant differences among treatments (*p* < 0.05; Waller–Duncan multiple comparison test following one-way ANOVA).

As shown in Figure 5 and Figure 6, aboveground P accumulation increased with crop development during the maize season, and significant differences among treatments were observed at key growth stages. Compared with CF, CF+CRB significantly increased P accumulation by 48.1% at V6 and by 14.5% at R6. In contrast, CF reached peak P accumulation at V12 and R4. Phosphorus partitioning showed a clear shift from vegetative organs to grains. At V12, P was mainly allocated to leaves, whereas the grain proportion increased to 66.41–78.46% at maturity.

During the soybean season, CF+CRB significantly increased P accumulation relative to CF by 168.7% at V1, 46.6% at R4, and 54.7% at R6, while CF+CRA achieved the highest P accumulation at R2 and R8. Phosphorus allocation also shifted from vegetative organs to grains, with grain P accounting for ≥82.83% at R8 across all treatments. Overall, CF+CRB resulted in higher P accumulation at early maize growth (V6) and at multiple soybean stages, whereas CF+CRA showed higher P accumulation at soybean R2 and R8. Across treatments, P partitioning consistently shifted from vegetative organs to grains, and grains served as the primary P pool at maturity.

### 2.5. Effects of Chemical Fertilizer Combined with Cattle Manure or Microbial Conditioners on Aboveground Potassium Accumulation of Maize and Soybean in a Maize–Soybean Rotation System

**Figure 7:**
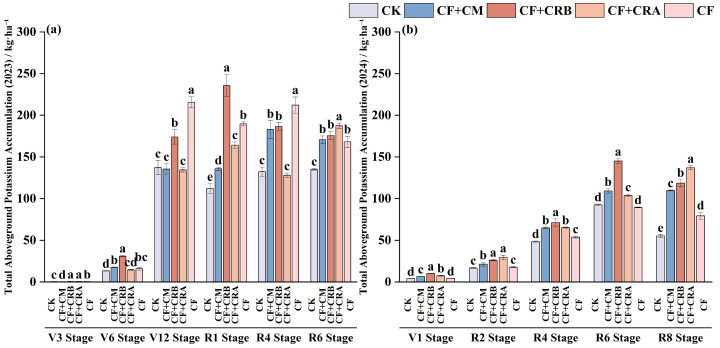
Effects of Combined Application of Cattle Manure and Microbial Amendments with Chemical Fertilizers on Aboveground Total Potassium Accumulation in a Maize–Soybean Rotation System: (**a**) Maize in the 2023 season; (**b**) Soybean in the 2024 season. Treatments: CK (control); CF (chemical fertilizer); CF+CM (CF + cattle manure); CF+CRA (CF + Complex Conditioner A); CF+CRB (CF + Complex Conditioner B). Values are presented as the mean ± standard deviation (SD) of three replicates (*n* = 3). Error bars indicate SD. Different lowercase letters above bars within the same panel indicate significant differences among treatments (*p* < 0.05; Waller–Duncan multiple comparison test following one-way ANOVA).

**Figure 8:**
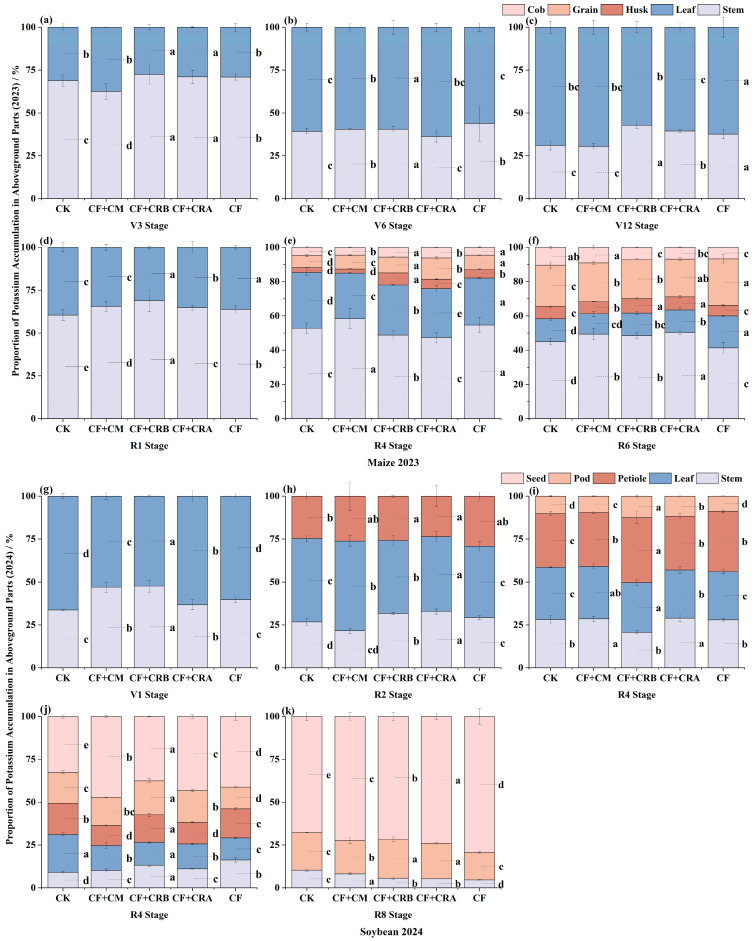
Effects of Combined Application of Cattle Manure and Microbial Amendments with Chemical Fertilizers on the Proportion of Potassium Accumulation in Different Aboveground Plant Parts in a Maize–Soybean Rotation System: (**a**) Maize at the V3 stage in 2023; (**b**) Maize at the V6 stage in 2023; (**c**) Maize at the V12 stage in 2023; (**d**) Maize at the R1 stage in 2023; (**e**) Maize at the R4 stage in 2023; (**f**) Maize at the R6 stage in 2023; (**g**) Soybean at the V1 stage in 2024; (**h**) Soybean at the R2 stage in 2024; (**i**) Soybean at the R4 stage in 2024; (**j**) Soybean at the R6 stage in 2024; (**k**) Soybean at the R8 stage in 2024. **Note:** Soybean has no seed tissue at the V1, R2, and R4 stages; therefore, the seed (grain) category is not shown in these panels and only appears from R6 and R8 onward. Treatments: CK (control); CF (chemical fertilizer); CF+CM (CF + cattle manure); CF+CRA (CF + Complex Conditioner A); CF+CRB (CF + Complex Conditioner B). Values are presented as the mean ± standard deviation (SD) of three replicates (*n* = 3). Error bars indicate SD. Different lowercase letters above bars within the same panel indicate significant differences among treatments (*p* < 0.05; Waller–Duncan multiple comparison test following one-way ANOVA).

As shown in Figure 7 and Figure 8, aboveground potassium (K) accumulation in maize and soybean varied over the growing season, and fertilization treatments significantly altered K uptake and partitioning. Overall, CF+CRB increased K accumulation at key stages and improved K allocation among organs.

In maize season, K accumulation differed significantly among treatments. At V3, CF+CRB and CF+CRA showed the highest K accumulation, which was 23.9% and 22.5% higher than CF, respectively. At V6, K accumulation under CF+CRB reached 30.91 kg·ha^−1^, representing a 94.9% increase relative to CF. At V12, CF exhibited the highest K accumulation (215.55 kg·ha^−1^). At R1, K accumulation under CF+CRB was 24.2% higher than that under CF, whereas at R6, CF+CRA had the highest K accumulation (187.45 kg·ha^−1^). K partitioning indicated a developmental shift from vegetative to reproductive organs. At V3, K was mainly distributed in stems and leaves (e.g., under CF+CRB, stems and leaves accounted for 72.6% and 27.4% of aboveground K, respectively). As development progressed, a greater proportion of K was allocated to grain; at R6, grain accounted for 27.1% of aboveground K under CF. In addition, CF+CRB significantly increased K accumulation in husk leaves at R4 (13.06 kg·ha^−1^), which was 22.3% higher than CF, indicating enhanced K translocation to reproductive tissues.

In soybean season, at V1, CF+CRB increased K accumulation by 143.1% relative to CF. At R2, CF+CRA and CF+CRB showed the highest K accumulation, exceeding CF by 65.3% and 46.5%, respectively. At R4, K accumulation under CF+CRB reached 71.13 kg·ha^−1^, a 32.4% increase over CF. At R6, CF+CRB remained the highest (144.97 kg·ha^−1^), whereas at R8, CF+CRA reached 136.97 kg·ha^−1^, suggesting stronger late-season K retention. K allocation also shifted from vegetative organs (stems and leaves) to reproductive organs (pods and seeds). At R2, stem and leaf K proportions were 30.2% and 43.6%, respectively, under CF+CRA. By R8, the seed K proportion increased markedly (e.g., 73.9% under CF+CRA), indicating efficient allocation to seeds. Moreover, CF+CRB significantly increased pod K accumulation at R6 (28.74 kg·ha^−1^), which was 153.6% higher than CF, further supporting its role in promoting K translocation to reproductive organs.

### 2.6. Effects of Chemical Fertilizer Combined with Cattle Manure or Microbial Conditioners on Nitrogen and Phosphorus Translocation in Maize Within a Maize–Soybean Rotation System

As shown in Table 1, fertilization treatments significantly altered maize N and P dynamics during grain formation, including pre-anthesis storage, post-anthesis uptake, and the relative contributions of these sources to grain accumulation.

The CF+CRB treatment showed the highest nitrogen translocation amount (NT, 39.90 kg·ha^−1^) and nitrogen translocation rate (NTR, 32.10%), both significantly higher than those under CF. Because NT and NTR quantify the net contribution of pre-anthesis N reserves to grain N accumulation, these higher values suggest greater reliance on remobilization of pre-anthesis stored N to grains under CF+CRB. In contrast, CF+CRA had significantly lower NT and NTR than CF. Notably, CK and CF+CM showed negative NT values, suggesting net N accumulation in vegetative organs after anthesis. This may reflect insufficient translocation driving forces or preferential allocation of post-anthesis absorbed N to vegetative tissues. Post-anthesis N accumulation (PNA) was highest under CF+CM (169.79 kg·ha^−1^), representing a 59.6% increase relative to CF, indicating that grain N supply in this treatment relied more strongly on post-anthesis uptake.

The CK treatment had the highest phosphorus translocation rate (PTR) and the highest contribution of P translocation to grain P (P-GCR). This pattern may reflect limited P availability, where a higher PTR primarily indicates internal redistribution (potentially passive) rather than a greater effective contribution to grain P accumulation. All fertilized treatments significantly increased post-anthesis P accumulation (PPA), with the highest values observed under CF+CRA (37.16 kg·ha^−1^) and CF+CRB (35.87 kg·ha^−1^), indicating enhanced P acquisition after anthesis under combined applications. Meanwhile, CF+CRB showed relatively lower PTR and P-GCR, which may be associated with increased post-anthesis P uptake, thereby reducing reliance on remobilization of P from vegetative organs.

Overall, the combined treatments improved grain formation efficiency by differentially regulating N and P translocation processes. CF+CRB was particularly effective in enhancing pre-anthesis N remobilization and post-anthesis P uptake, whereas CF+CM mainly increased post-anthesis N supply.

### 2.7. Effects of Chemical Fertilizer Combined with Cattle Manure or Microbial Conditioners on Nitrogen and Phosphorus Use Efficiency in Maize Within a Maize–Soybean Rotation System

As shown in Table 2, fertilization treatments significantly affected maize N and P use efficiency, nutrient balance, and harvest indices.

The CF+CRB treatment achieved the highest NUE, AEN, and PFP_N_ and differed significantly from the other fertilized treatments. Relative to CF, CF+CRB increased NUE, AEN, and PFP_N_ by 58.4%, 114.1%, and 18.9%, respectively (calculated from Table 2). Nitrogen balance differed markedly among treatments: CF+CRB showed the lowest N surplus/deficit (40.43 kg·ha^−1^), whereas CF+CRA showed the highest (123.36 kg·ha^−1^). The nitrogen harvest index (NHI) was highest under CF+CM, which was 6.4% higher than the lowest treatment (CK), and fertilized treatments generally showed higher NHI than CK.

The CF+CRB treatment also showed the highest phosphorus use efficiency (PUE), agronomic efficiency of P (PAE), and partial factor productivity of P (PFP_P_), all significantly higher than the other treatments. Compared with CF, CF+CRB increased PUE by 37.8%, PAE by 114.3%, and PFP_P_ by 18.9%. Phosphorus balance differed significantly among treatments: CF+CRA had the highest P surplus/deficit (77.65 kg·ha^−1^), whereas CF+CRB had the lowest. The phosphorus harvest index (PHI) varied substantially across treatments, with relatively higher values under CK and CF+CM, intermediate values under CF+CRB, and lower values under CF and CF+CRA. In particular, PHI under CF+CM was 18.1% higher than that under CF+CRA.

Overall, the combined treatments improved fertilizer use through different pathways. CF+CRB consistently performed best in N and P use efficiency, agronomic efficiency, and partial factor productivity, indicating superior synergistic improvements in nutrient uptake and conversion. In contrast, CF+CM showed the highest NHI and PHI, suggesting enhanced allocation of absorbed nutrients to grain.

### 2.8. Effects of Chemical Fertilizer Combined with Cattle Manure or Microbial Conditioners on Apparent N and P Use Efficiency in Soybean Under a Maize–Soybean Rotation System

As shown in Table 3, the combined treatments significantly affected the apparent use efficiency of N and P fertilizers in soybean.

The CF+CRB treatment performed best, with an apparent N recovery (ANR) of 184.84%, which was significantly higher than all other treatments. CF+CM showed an ANR of 102.11%, whereas CF+CRA was 99.96%; CF was the lowest. An ANR exceeding 100% suggests that the increase in plant N uptake cannot be explained solely by in-season N fertilization and may also involve reutilization of residual soil N, soil N mineralization, and differences in biological N fixation among treatments. Agronomic efficiency of N (AEN) was also highest under CF+CRB (28.75 kg·kg^−1^), representing a 142.00% increase compared with CF. The CF+CM and CF+CRA treatments achieved 15.16 and 16.33 kg·kg^−1^, corresponding to increases of 27.61% and 37.46% relative to CF, respectively. Partial factor productivity of N (PFP_N_) reached 50.16 kg·kg^−1^ under CF+CRB, 79.85% higher than CF, whereas CF+CM (30.11 kg·kg^−1^) and CF+CRA (26.78 kg·kg^−1^) did not differ significantly from CF. The nitrogen harvest index (NHI) was generally high, with the highest value under CF and the lowest under CF+CM.

The CF+CRB treatment had the highest apparent P recovery (APR, 28.79%), an 85.50% increase relative to CF. CF+CRA also increased APR to 19.78%, which was 27.45% higher than CF, while CF+CM did not differ significantly from CF. Agronomic efficiency of P (PAE) was highest under CF+CRB (25.39 kg·kg^−1^), 90.33% higher than CF. CF+CRA did not differ significantly from CF, whereas CF+CM was significantly lower than CF. Partial factor productivity of P (PFP_P_) under CF+CRB reached 44.30 kg·kg^−1^, 41.44% higher than CF. In contrast, CF+CM (24.43 kg·kg^−1^) and CF+CRA (22.51 kg·kg^−1^) were significantly lower than CF. The P harvest index (PHI) was highest under CF+CM, 10.66% higher than CF. CF+CRB and CF+CRA showed PHI values of 86.00% and 85.64%, which were 3.18% and 2.82% higher than CF, respectively.

Overall, the combined treatments improved soybean nutrient use efficiency through different pathways. CF+CRB consistently performed best in apparent recovery, agronomic efficiency, and partial factor productivity for both N and P, highlighting the integrated advantage of microbial inoculation in promoting nutrient uptake. In contrast, CF+CM primarily increased PHI, indicating enhanced allocation of P to grains.

### 2.9. Effects of Combined Application of Cattle Manure and Microbial Amendments with Fertilizers on Yield in a Maize–Soybean Rotation System

**Figure 9:**
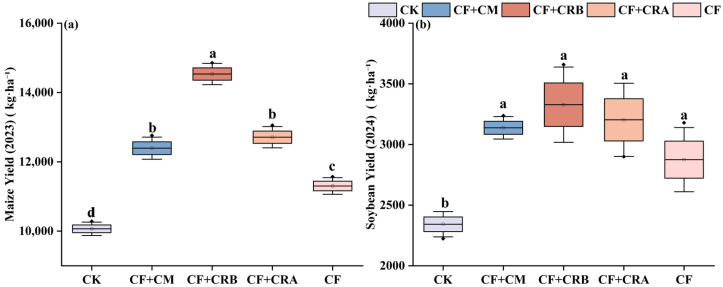
Effects of Combined Application of Cattle Manure, Microbial Amendments, and Chemical Fertilizers on Crop Yield in a Maize–Soybean Rotation System: (**a**) Maize yield in 2023; (**b**) Soybean yield in 2024. **Note:** Treatments: CK (control); CF (chemical fertilizer); CF+CM (CF + cattle manure); CF+CRA (CF + Complex Conditioner A); CF+CRB (CF + Complex Conditioner B). Values are presented as the mean of three replicates (*n* = 3). Error bars indicate SD. Different lowercase letters above bars within the same panel indicate significant differences among treatments (*p* < 0.05; Waller–Duncan multiple comparison test following one-way ANOVA).

As shown in Figure 9, fertilization treatments significantly affected grain yield in the maize–soybean rotation system.

Maize season. Compared with CF, all combined treatments increased grain yield. The largest increase was observed under CF+CRB, reaching 14,533.13 kg·ha^−1^, which was 28.6% higher than CF. Grain yield under CF+CRA and CF+CM was 12,710.84 and 12,394.04 kg·ha^−1^, representing increases of 12.5% and 9.6% relative to CF, respectively, with no significant difference between these two treatments.

Soybean season. Seed yield was highest under CF+CRB (3328.15 kg·ha^−1^), which was 15.8% higher than CF. Yields under CF+CM (3137.55 kg·ha^−1^) and CF+CRA (3203.25 kg·ha^−1^) were 9.1% and 11.4% higher than CF, respectively, and did not differ significantly from CF+CRB.

Overall, the combined treatments enhanced system productivity primarily by increasing grain yield. CF+CRB significantly outperformed CF in both seasons, increasing maize and soybean yields by 28.6% and 15.8%, respectively, and achieved the highest grain yield among all treatments.

### 2.10. Spearman Correlation and Mantel Test of Crop System Associations

**Figure 10:**
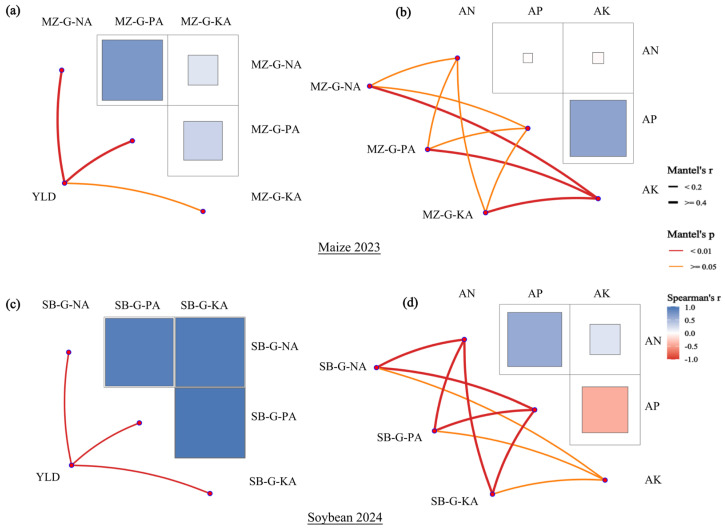
Mantel test and Spearman correlation analyses of soil available nutrients, grain nutrient accumulation, and yield traits under different fertilization treatments in a maize–soybean rotation system. (**a**) Relationships among maize yield and grain N, P, and K accumulation in 2023. (**b**) Relationships between maize grain N, P, and K accumulation and soil alkali-hydrolyzable N, available P, and available K in 2023. (**c**) Relationships among soybean yield and grain N, P, and K accumulation in 2024. (**d**) Relationships between soybean grain N, P, and K accumulation and soil alkali-hydrolyzable N, available P, and available K in 2024. **Note:** The upper and lower panels represent maize (2023) and soybean (2024), respectively. Colored edges indicate Mantel test results: edge color denotes significance (red, *p* < 0.01; orange, 0.01 ≤ *p* < 0.05; gray, *p* ≥ 0.05), and edge width denotes Mantel’s r (|r| < 0.2, thin; |r| ≥ 0.4, thick). Heatmap cells show Spearman’s rank correlation coefficients (ρ), with blue indicating positive correlations and red indicating negative correlations (color intensity reflects the magnitude). Abbreviations: YLD, yield; AN, alkali-hydrolyzable N; AP, available P; AK, available K; MZ-G-NA/PA/KA, maize grain N/P/K accumulation; SB-G-NA/PA/KA, soybean grain N/P/K accumulation.

As shown in Figure 10, during the maize season, Spearman correlations among soil nutrient indicators revealed a significant positive association between available P (AP) and available K (AK). The Mantel test further indicated that AK was significantly associated with grain N, P, and K accumulation in maize. This suggests a close statistical linkage between soil K availability and nutrient accumulation in maize grain. Maize yield (YLD) was highly and positively correlated with grain N and P accumulation, with the strongest association observed between yield and grain P accumulation.

During the soybean season, the correlation structure among soil nutrients differed from that in maize. Spearman correlations showed a positive relationship between soil alkali-hydrolyzable N (AN) and AP, indicating that available N and P supply tended to co-vary across samples. The Mantel test further showed that both AN and AP were significantly associated with soybean N, P, and K accumulation, with some associations being relatively strong. This highlights a more prominent statistical linkage between soybean nutrient accumulation and the status of soil available N and P. Meanwhile, grain N, P, and K accumulation in soybean were highly coordinated with each other, and all three were highly positively correlated with yield. This indicates that multi-element accumulation in soybean grain often increased synchronously and was jointly associated with yield.

Taken together, these results demonstrate season-dependent association patterns between soil available nutrients and crop nutrient accumulation/yield within the rotation system. The maize season emphasized the centralized association of AK with multi-element accumulation in grain, whereas the soybean season showed more synchronous associations of AN and AP with nutrient accumulation. In both seasons, yield was consistently positively associated with grain nutrient accumulation, but the coordination among grain N, P, and K accumulation was stronger in soybean.

## 3. Discussion

Within the maize–soybean rotation in Albic soils, our results highlight that soil nutrient availability is not static but varies markedly with crop phenology, and these within-season shifts appear to differentiate the performance of the integrated fertilization strategies. In particular, the pronounced stage-dependent changes in alkali-hydrolyzable N, available P and available K (Figure 1) indicate that the effectiveness of a given amendment depends on whether it can increase nutrient supply at the time when crop demand is highest, rather than simply increasing nutrient levels on average. Similar phenology-linked spatiotemporal variability in soil nutrients and corresponding crop responses has been reported in field systems, underscoring the agronomic relevance of synchronizing nutrient supply with growth-stage demand [36]. This feature is particularly relevant in Albic soils, where limited nutrient buffering and retention can amplify within-season fluctuations in available nutrients, increasing the risk of transient mismatches between nutrient supply and crop demand and thereby reducing fertilizer use efficiency [37].

The treatment comparisons further suggest that different amendments regulate nutrient supply through distinct, nutrient-specific pathways. CF+CRA markedly elevated soil available P during the soybean flowering–pod stage, aligning with the reproductive-phase requirement for rapid P acquisition and suggesting enhanced P mobilization in situ; sustained fertilization strategies are known to influence P availability and resupply dynamics during crop growth [38], and yield benefits from organic or integrated amendments are frequently mediated through microbially driven improvements in soil functioning [39]. The increase in available P under CF+CRA is also consistent with functions attributed to phosphate-solubilizing microorganisms (e.g., transformation of less-available P pools and rhizosphere mobilization), which can promote plant P uptake [40]. By contrast, CF+CM maintained relatively higher available K at soybean maturity, consistent with a more sustained nutrient release and/or improved nutrient retention associated with manure inputs; long-term cow manure combined with inorganic fertilization has been shown to enhance soil fertility and support yield formation, implying prolonged nutrient supply effects [41]. Notably, CF+CRB rarely produced the single highest value for any nutrient at an individual stage; however, it maintained comparatively balanced levels of alkali-hydrolyzable N, available P and available K across several key stages. This pattern implies that CF+CRB may reduce the amplitude of nutrient “peaks and troughs” over the rotation cycle, thereby providing a more stable nutrient supply environment for crop uptake.

Dry matter accumulation is a key factor influencing yield formation and serves as an important indicator for evaluating the effectiveness of fertilization strategies. In this study, CF+CRB produced the highest dry matter accumulation at maize V6 and R1, as well as at soybean V1 and R4, indicating that this treatment strengthened early crop vigor and enhanced assimilate accumulation during mid-to-late growth. This advantage was consistent with its superior N and P uptake. In particular, at maize maturity, N and P accumulation under CF+CRB was significantly higher than that under CF, providing a stronger nutrient foundation for yield formation.

Previous studies have shown that adding organic amendments can increase soil organic matter and the supply of mineralizable nutrients, thereby modifying the release patterns and sustained availability of N, P, and K. As a result, the within-season supply dynamics of soil alkali-hydrolyzable N, available P, and available K can be substantially altered [42]. Microbial inoculants can further enhance this process by colonizing the rhizosphere and releasing metabolites that directly mobilize nutrients and stimulate root uptake, thereby improving nutrient transfer efficiency from soil to crop at the system level [43,44].

Crop yield depends not only on nutrient uptake but also on the efficiency of nutrient translocation and partitioning within the plant. During the maize season, CF+CRB showed the highest pre-anthesis N translocation amount and translocation rate. This pattern suggests that the treatment may promote early N accumulation in vegetative organs and enable more effective remobilization to grains during grain filling, suggesting a pattern of pre-anthesis storage followed by late translocation [45,46]. In contrast, CF+CM exhibited a negative N translocation value, indicating no net N export from vegetative organs between anthesis and harvest. Under this treatment, grain N formation may rely more on post-anthesis N uptake, consistent with the sustained N release associated with manure mineralization [47,48].

For P, CF+CRB had the highest post-anthesis P uptake but a lower P translocation rate, indicating that plants maintained a strong capacity to acquire P from soil during late growth. This may reduce dependence on P remobilization from vegetative tissues, help sustain leaf function, and support grain filling [49,50].

With respect to K utilization, the CF+CRB treatment showed clear advantages in K accumulation at maize V6 and R1, increasing it by 94.9% and 24.2%, respectively, compared with CF. This suggests that microbial inoculation may enhance early K uptake and promote K storage during vegetative growth [51]. In the soybean season, K accumulation under CF+CRB and CF+CRA was significantly higher than under CF at R2 and R4, with increases of 46.5, 65.3% and 32.4%, respectively, indicating that inoculation helps meet the high K demand during the flowering–pod stage. In addition, the organ partitioning pattern of K was consistent with that of N and P, showing a shift from vegetative organs to reproductive organs. For example, CF+CRB significantly increased K accumulation in maize husk leaves at R4 (by 22.3% relative to CF) and in soybean pods at R6 (by 153.6%), further indicating that the combined treatments may support yield formation by optimizing K allocation to grain [52].

Fertilizer use efficiency and nutrient balance further indicate a potential advantage of integrated fertilization in improving resource use while reducing the risk of residual nutrient accumulation. In the maize season, CF+CRB showed significantly higher N and P use efficiency than CF (26.19% and 26.68%, respectively) and also exhibited the lowest N and P surplus/deficit values. This suggests that yield gains under CF+CRB were achieved with lower risks of nutrient carryover and loss. In the soybean season, the apparent N recovery of all combined treatments exceeded 100%. Because soybean can fix atmospheric N, fertilization and combined amendments may modify the rhizosphere N environment and thereby influence the intensity of symbiotic N fixation, which in turn alters total plant N uptake. In addition, previous studies have reported a positive priming effect of N inputs: small amounts of mineral N can reduce the soil C:N ratio and stimulate microbial decomposition of organic matter, leading to the release of substantial amounts of soil organic N [53]. Together with residual N from the preceding crop and in-season mineralization under crop rotation, these processes can result in apparent recovery values greater than 100% [54,55,56]. Importantly, this pattern is also related to the accounting framework used to calculate apparent recovery in this study, which incorporates contributions from prior-crop nutrient carryover and the soil nutrient pool. Therefore, ANR > 100% reflects enhanced utilization of residual soil nutrients and supports the view that manure and microbial inoculants can strengthen nutrient cycling in rotation systems [54,57]. Overall, appropriately combining organic amendments or microbial inoculants with chemical fertilizers can improve the sustainability of nutrient use.

In this study, the combined treatments showed distinct effects on yield, indicating treatment-specific patterns in productivity improvement. CF+CRB treatment increased maize and soybean yields by 28.6% and 15.8%, respectively, demonstrating a significant yield advantage across the entire rotation cycle.

## 4. Materials and Methods

### 4.1. Experimental Site

**Figure 11:**
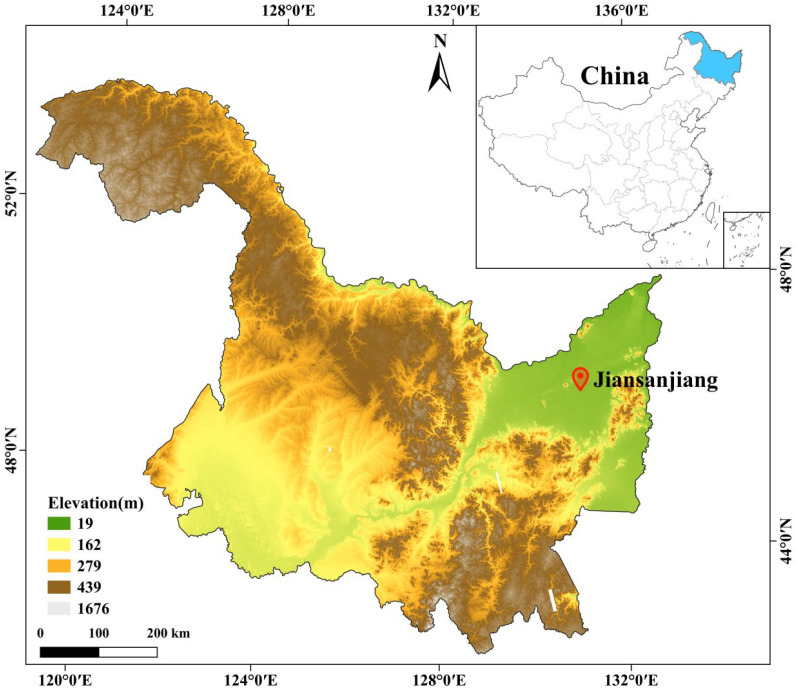
Geographical location of the experimental site.

**Figure 12:**
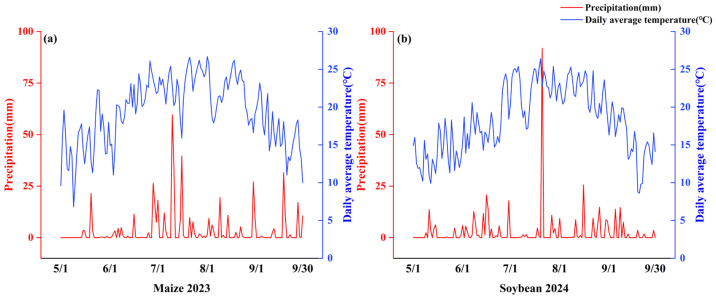
Daily mean air temperature (°C) and precipitation (mm) during the maize (2023) (**a**) and soybean (2024) (**b**) growing seasons.

The field experiment was conducted in 2023–2024 at the Sixth Management District of Qixing Farm, Jiansanjiang City, Jiamusi, Heilongjiang Province, China (132°35′58.402″ E, 47°10′54.401″ N). The site is located on an alluvial-lacustrine low plain of the Sanjiang Plain and is characterized by the presence of Albic soils. The region has a cold-temperate, humid monsoon climate, with a long-term mean annual temperature of 3.2 °C and mean annual precipitation of 550–600 mm. Figure 11 is the geographical location of the experimental site, Figure 12 is the daily average temperature and precipitation during the growing season of maize 2023 and soybean 2024.

### 4.2. Experimental Design

The maize cultivar Lihe 328 was provided by Limagrain Specialty Grains R&D Co., Ltd. (Shanxi, China). The soybean cultivar Suinong 52 was provided by the Suihua Branch of Heilongjiang Academy of Agricultural Sciences (Heilongjiang, China). During the maize season in 2023, the chemical fertilizer used was Beifeng Hanfeng maize fertilizer (N:P:K = 26:12:12). During the soybean season in 2024, the chemical fertilizer used was Sinochem Fuwannong blended fertilizer (N:P:K = 13:25:12). The organic fertilizer was purchased from Heilongjiang Heiwotu Biotechnology Co., Ltd. (Heilongjiang, China). Complex Conditioner A (CRA) was a microbial fertilizer marketed as Jiaqifeng microbial fertilizer, and Complex Conditioner B (CRB) was a microbial inoculant marketed as Heiwotu microbial inoculant. The nutrient contents of the organic materials are reported in Table 4, and baseline soil fertility is summarized in Table 5. The 2024 baseline soil properties were determined from samples collected after the 2023 maize harvest and prior to 2024 soybean sowing, which reflect carryover effects from the preceding crop and fertilization history.

The experiment used a maize–soybean rotation system. Land preparation consisted of autumn ridging. During the maize season, a wide-ridge double-row planting pattern was used, whereas a wide-ridge triple-row planting pattern was used during the soybean season. Organic amendments (cattle manure or microbial inoculants) were surface-broadcast after ridging and evenly applied to the surface layer of the furrows. They were subsequently incorporated into the 0–20 cm topsoil during tillage. Application rates of the organic amendments were selected based on locally optimized recommendations derived from multi-year field trials in Albic soils of Heilongjiang Province, China. The cattle manure (CM) product used in this study is a concentrated organic amendment (N–P–K = 1.5–1.0–1.2%, organic matter 28%). Therefore, comparisons of CM rates across regions should be interpreted with caution when expressed on a fresh-weight basis, because manure types (e.g., fresh FYM vs. composted/dewatered products) differ markedly in moisture content and nutrient concentration. For this reason, we focus on the nutrient input context of the applied amendment rather than making a direct mass-based comparison with other regions. This rate was intended to improve soil quality and nutrient cycling while minimizing environmental risks (e.g., nutrient leaching) in these vulnerable soils, and it provides a moderate nutrient input given the manure composition. The application rates of the microbial amendments were further adjusted based on preliminary plot trials at the study site to ensure effective rhizosphere establishment under local soil conditions. The lower rate of CRB was used because of its higher viable cell density, whereas CRA was applied at a higher rate to maintain adequate inoculum supply and functional persistence in the rhizosphere. Maize was sown on 28 April 2023 and harvested on 15 September 2023. Soybean was sown on 6 May 2024 and harvested on 1 October 2024. Irrigation, weed control, and pest and disease management followed local standard practices throughout each growing season.

The experiment was arranged in a randomized complete block design with five treatments and three replicates, resulting in 15 plots. Each plot was 50 m long and consisted of six rows, with a ridge width of 1.1 m. Row spacing was 40 cm in the maize season and 22 cm in the soybean season. The application rates of chemical fertilizers and organic amendments for all treatments in the maize (2023) and soybean (2024) seasons are provided in Table 6. In the soybean season, only basal fertilizer was applied (no top-dressing) to isolate the effects of basal fertilization in combination with organic amendments.

### 4.3. Measurement Items and Methods

During the 2023 maize season, sampling was conducted at the seedling stage (V3), jointing stage (V6), 12-leaf stage (V12), silking stage (R1), grain-filling stage (R4), and physiological maturity (R6). During the 2024 soybean season, sampling was conducted at the trifoliate stage (V1), full flowering (R2), full pod (R4), seed-filling stage (R6), and maturity (R8). The experiment was arranged in a randomized complete block design with three replicates (*n* = 3). At each sampling time, three plants were randomly selected from each plot. The same tissue type (e.g., leaves) from the three plants was pooled to form one composite sample per plot for subsequent chemical analysis.

- Dry Matter Content and Nutrient Content in Plants;

Plant samples were separated by organ, and after being blanched at 105 °C for 30 min, they were dried at 80 °C to constant weight. The dry weight of each organ was measured using an electronic balance with a precision of 0.01 g.

The total nitrogen and total phosphorus content in the plant samples were determined using the H_2_SO_4_−H_2_O_2_ digestion method [58,59]. A sample of 0.2 g of the dried, sieved material was placed in a digestion tube, and 5 mL of concentrated sulfuric acid was added. The tube was left overnight to allow for complete acid digestion. The sample was then heated in a digestion furnace until white fumes appeared, followed by the slow addition of 30% H_2_O_2_ while raising the temperature. The digestion process continued until the solution was clear and transparent (digestion block, DK 20; VELP Scientifica, Rome, Italy). The digested solution was cooled, made up to 100 mL, and filtered for analysis. Total nitrogen content was determined by the Kjeldahl method, using a distillation apparatus (UDK 129; VELP Scientifica, Rome, Italy) to volatilize ammonia and absorb it in boric acid, and the nitrogen concentration was calculated by titration with standard hydrochloric acid. Total phosphorus content was measured using the vanadate–molybdate yellow colorimetric method. Part of the digested solution was reacted with vanadate–molybdate reagent to form a yellow complex, and the absorbance was measured at 420 nm using a UV–Vis spectrophotometer (UV-1800; Shimadzu, Japan). The phosphorus concentration was determined by comparison with a standard curve. Total potassium content was measured using a flame photometer (PFP7; Jenway, UK).

- Crop Yield Measurement;

Maize was harvested after maturity, and the ears were air-dried and threshed to determine yield. The sampling area was 5 m^2^, and the grain yield was adjusted to 14% moisture content. Soybean was harvested at maturity, air-dried, and threshed to determine yield. The sampling area was 5 m^2^, and the grain yield was adjusted to 13% moisture content.

- Calculation;

To quantify key indicators of nitrogen, phosphorus, and potassium absorption, transport, and fertilizer use efficiency, the following calculations were made for each organ’s nitrogen and phosphorus accumulation, nitrogen and phosphorus transport, transport rate, transport contribution to grain, post-flowering nutrient accumulation, harvest index, agronomic efficiency, fertilizer use efficiency, and fertilizer productivity [60,61,62,63].

The nitrogen accumulation formula for an organ (NAO) is as follows:

NAO = DM × NC,(1)

DM is the dry matter weight of each organ per hectare; NC is the nitrogen content percentage of each organ.

The nitrogen transport amount (NT) is calculated as follows:

NT = NA_V, FL_−NA_V, HA_, (2)

NA_V, FL_ is the nitrogen accumulation at flowering; NAV, HA is the nitrogen accumulation in the harvest period in the vegetative organs.

The nitrogen transport rate (NTR) is calculated as:

NTR = NT/NA_V, FL_, (3)

NT is the nitrogen transport amount; N_AV, FL_ is the nitrogen accumulation at flowering.

The nitrogen transport contribution to grain (N-GCR) is calculated as:

N-GCR = NT/NA_G, HA_, (4)

NT is the nitrogen transport amount; N_AG, HA_ is the nitrogen accumulation in grains at harvest.

The post-flowering nitrogen accumulation (PNA) is calculated as:

PNA = NA_p, HA_ − NA_p, FL_, (5)

NA_p, HA_ is the total nitrogen accumulation in the plant at harvest; NA_p, FL_ is the total nitrogen accumulation in the plant at flowering.

The nitrogen harvest index (NHI) is calculated as:

NHI = N_AG_/N_AS_, (6)

N_AG_ is the nitrogen absorbed by the grain; N_AS_ is the total nitrogen absorbed by the above-ground biomass.

The agronomic efficiency of nitrogen (AEN) is calculated as:

AEN = (Y_N_ − Y_0_)/N_a_, (7)

Y_N_ is the maize yield in the nitrogen application plot; Y_0_ is the maize yield in the non-nitrogen application plot; N_a_ is the nitrogen applied.

The nitrogen use efficiency (NUE) is calculated as:

NUE = (N_AN_ − N_A0_)/N_a_, (8)

N_AN_ is the nitrogen absorbed by the plants in the nitrogen application plot; N_A0_ is the nitrogen absorbed by the plants in the non-nitrogen application plot; N_a_ is the nitrogen applied.

The nitrogen fertilizer apparent recovery (ANR) is calculated as:

ANR = (N_AN_ − N_A0_)/(Na + N_carryover_), (9)

N_AN_ is the nitrogen absorbed by the plants in the nitrogen application plot; N_A0_ is the nitrogen absorbed by the plants in the non-nitrogen application plot; N_a_ is the nitrogen applied; N_carryover_ is the nitrogen surplus from the previous season.

The nitrogen fertilizer productivity (PFP_N_) is calculated as:

PFP_N_ = Y_G_/N_a_ (10)

Y_G_ is the grain yield; N_a_ is the nitrogen applied.

The calculation formulas for phosphorus-related indicators are the same as for nitrogen, and are therefore not repeated here.

- Soil chemical index;

Soil alkali-hydrolyzable nitrogen was determined using the alkali-diffusion method [64]. Available phosphorus was measured using the sodium bicarbonate extraction-molybdenum-antimony colorimetric method [64]. Available potassium was measured using the ammonium acetate extraction-flame photometry method [65].

### 4.4. Data Processing

The experimental data were pre-processed using Excel 2021 (Microsoft, Redmond, WA, USA). Normality and homogeneity of variances were assessed using the Shapiro–Wilk test and Levene’s test, respectively. When assumptions were violated, data were log- or square-root-transformed prior to ANOVA. One-way ANOVA was performed in SPSS 26.0 (IBM, Chicago, IL, USA), and treatment means were compared using Duncan’s multiple range test at α = 0.05. Graphs were created using Origin 2024 (OriginLab, Northampton, MA, USA). Mantel tests and Spearman correlation analysis were used to evaluate relationships among crop N, P, and K uptake, yield, and soil available nutrient indicators.

## 5. Conclusions

In the maize season, combining chemical fertilizer with microbial inoculants or cattle manure can significantly improve nutrient acquisition and utilization processes and increase yield. Compared with chemical fertilizer alone, co-application of the CRB inoculant enhanced pre-anthesis N translocation and post-anthesis P uptake in maize, resulting in concurrent improvements in N and P fertilizer use efficiency and a significant yield increase (maize grain yield reached 14,533 kg·ha^−1^). In the soybean season, the CRB and CRA inoculants promoted nutrient uptake during key reproductive stages, whereas cattle manure was more favorable for sustaining nutrient supply during late growth, particularly by maintaining soil available K. Overall, integrated management in rotation systems should be differentiated by crop season and target traits: CRB is recommended in the maize season to improve nutrient use efficiency and yield, whereas in the soybean season, cattle manure or a phosphate-oriented inoculant can be selected to optimize the supply of key nutrients according to production goals. This strategy provides a theoretical reference for reduced-input, higher-efficiency management and green agricultural development of maize–soybean rotation systems in Albic soil regions.

## Figures and Tables

**Table 1:** Effects of Combined Application of Cattle Manure, Microbial Amendments, and Chemical Fertilizers on Nitrogen and Phosphorus Translocation Characteristics of Maize in a Maize–Soybean Rotation System.

Treatment	CK	CF+CM	CF+CRB	CF+CRA	CF
NT (kg·ha^−1^)	−2.13 ^c^	−12.46 ^c^	39.90 ^a^	17.48 ^b^	29.23 ^a,b^
NTR (%)	−4.14 ^c^	−19.59 ^d^	32.10 ^a^	17.86 ^b^	28.78 ^a,b^
N-GCR (%)	−2.11 ^c^	−8.60 ^c^	28.83 ^a^	12.18 ^b^	23.37 ^a^
PNA (kg·ha^−1^)	107.25 ^c^	169.79 ^a^	111.47 ^c^	143.50 ^b^	106.41 ^c^
PT (kg·ha^−1^)	0.57 ^a^	0.38 ^c^	0.20 ^d^	0.43 ^b^	0.37 ^c^
PTR (%)	78.44 ^a^	69.75 ^b^	47.73 ^e^	62.55 ^c^	57.56 ^d^
P-GCR (%)	3.76 ^a^	1.53 ^b^	0.72 ^c^	1.72 ^b^	1.73 ^b^
PPA (kg·ha^−1^)	18.75 ^c^	30.71 ^b^	35.87 ^a^	37.16 ^a^	31.03 ^b^

**Note:** NT (nitrogen transport amount); NTR (nitrogen transport rate); N-GCR (nitrogen transport contribution to grain); PNA (post-flowering nitrogen accumulation); PT (phosphorus transport amount); PTR (phosphorus transport rate); P-GCR (phosphorus transport contribution to grain); PPA (post-flowering phosphorus accumulation). Values are presented as the mean of three replicates (*n* = 3). Error bars indicate SD. Different lowercase letters above bars within the same panel indicate significant differences among treatments (*p* < 0.05; Waller–Duncan multiple comparison test following one-way ANOVA).

**Table 2:** Effects of Combined Application of Cattle Manure, Microbial Amendments, and Chemical Fertilizers on Nitrogen and Phosphorus Use Efficiency of Maize in a Maize–Soybean Rotation System.

Treatment	CK	CF+CM	CF+CRB	CF+CRA	CF
NUE (%)	-	23.68 ^b^	26.19 ^a^	21.66 ^b^	16.53 ^c^
AEN (kg·kg^−1^)	-	8.09 ^b^	16.21 ^a^	9.10 ^b^	7.57 ^b^
PFP_N_ (kg·kg^−1^)	-	42.07 ^c^	54.37 ^a^	37.84 ^d^	45.73 ^b^
Nitrogen surplus (kg·ha^−1^)	-	74.50 ^b^	40.43 ^d^	123.36 ^a^	66.93 ^c^
NHI	58.17 ^c^	61.87 ^a^	59.37 ^b,c^	59.42 ^b,c^	60.22 ^b^
PUE (%)	-	13.77 ^c^	26.68 ^a^	15.91 ^b,c^	19.36 ^b^
PAE (kg·kg^−1^)	-	29.16 ^b^	70.64 ^a^	28.73 ^b^	32.96 ^b^
PFP_P_ (kg·kg^−1^)	-	151.68 ^c^	236.91 ^a^	119.42 ^d^	199.23 ^b^

**Note:** NUE (nitrogen use efficiency); AEN (agronomic efficiency of nitrogen); PFPN (partial factor productivity of nitrogen); NHI (nitrogen harvest index); PUE (phosphorus use efficiency); PAE (agronomic efficiency of phosphorus); PFPP (partial factor productivity of phosphorus). Values are presented as the mean of three replicates (*n* = 3). Error bars indicate SD. Different lowercase letters above bars within the same panel indicate significant differences among treatments (*p* < 0.05; Waller–Duncan multiple comparison test following one-way ANOVA).

**Table 3:** Effects of Combined Application of Cattle Manure, Microbial Amendments, and Chemical Fertilizers on Apparent Nitrogen and Phosphorus Recovery in Soybean in a Maize–Soybean Rotation System.

Treatment	CK	CF+CM	CF+CRB	CF+CRA	CF
ANR (%)	-	102.11 ^b^	184.84 ^a^	99.96 ^b^	62.03 ^c^
AEN (kg·kg^−1^)	-	15.16 ^b^	28.75 ^a^	16.33 ^b^	11.88 ^c^
PFP_N_ (kg·kg^−1^)	-	30.11 ^b^	50.16 ^a^	26.78 ^b^	27.89 ^b^
(NHI)	94.91 ^b^	92.89 ^d^	93.65 ^c^	95.05 ^a,b^	95.47 ^a^
APR (%)	-	15.00 ^c^	28.79 ^a^	19.78 ^b^	15.52 ^c^
PAE (kg·kg^−1^)	-	12.30 ^c^	25.39 ^a^	13.73 ^b^	13.34 ^b^
PFP_P_ (kg·kg^−1^)	-	24.43 ^c^	44.30 ^a^	22.51 ^d^	31.32 ^b^
(PHI)	85.08 ^b^	93.48 ^a^	86.00 ^b^	85.64 ^b^	82.82 ^c^

**Note:** ANR (apparent nitrogen recovery); AEN (agronomic efficiency of nitrogen); PFPN (partial factor productivity of nitrogen); NHI (nitrogen harvest index); APR (apparent phosphorus recovery); PAE (agronomic efficiency of phosphorus); PFPP (partial factor productivity of phosphorus); PHI (phosphorus harvest index). The values used to calculate the apparent nutrient recovery rates in the table are the fertilization amounts applied during the soybean season, plus the nutrient surplus from the previous year. The potassium application during the soybean season is relatively low. Values are presented as the mean of three replicates (*n* = 3). Error bars indicate SD. Different lowercase letters above bars within the same panel indicate significant differences among treatments (*p* < 0.05; Waller–Duncan multiple comparison test following one-way ANOVA).

**Table 4:** Basic characteristics of cattle manure and Complex Conditioners used in the experiment.

	Cattle Manure (kg·ha^−1^)	Complex Conditioner A (kg·ha^−1^)	Complex Conditioner B (kg·ha^−1^)
Content of N, P, K nutrients (%)	1.5, 1.0, 1.2	12, 7, 6	Not detected
Organic matter content (%)	28	20	20
Microbial community composition	No data	*Bacillus subtilis*, *B. licheniformis*, *B. amyloliquefaciens* ≥ 0.2 × 10^8^ CFU·g^−1^	*Bacillus subtilis*, *B. licheniformis*, *B. megaterium* ≥ 5 × 10^8^ CFU·g^−1^

**Table 5:** Basic physicochemical properties of the soil (0–20 cm) before the experiment in 2023 (pre-maize) and in 2024 (post-maize, pre-soybean).

Year	pH	Alkali-Hydrolyzable Nitrogen (mg·kg^−1^)	Available Phosphorus (mg·kg^−1^)	Available Potassium (mg·kg^−1^)	Organic Carbon (g·kg^−1^)	Total Nitrogen (g·kg^−1^)
2023	7.42	112.69	23.52	213.91	41.84	3.36
2024	7.59	102.67	23.32	199.89	36.39	3.03

**Table 6:** Detailed fertilization scheme for different treatments in the maize–soybean rotation field experiment (2023–2024).

Treatment	Maize 2023			Soybean 2024
	Basal Fertilizer (kg·ha^−1^)	Organic Matter Addition (kg·ha^−1^)	Topdressing (Urea) (kg·ha^−1^)	Basal Fertilizer kg·ha^−1^
CK	0	0	0	0
CF	525	0	300	300
CF+CM	525	Cattle manure: 2250 kg·ha^−1^	300	300
CF+CRA	525	Complex Conditioner A: 750 kg·ha^−1^	300	300
CF+CRB	525	Complex Conditioner B: 300 kg·ha^−1^	300	300

**Note:** Treatments: CK (control); CF (chemical fertilizer); CF+CM (CF + cattle manure); CF+CRA (CF + Complex Conditioner A); CF+CRB (CF + Complex Conditioner B).

## Data Availability

The original contributions presented in this study are included in the article. Further inquiries can be directed to the corresponding author.

## Abbreviations

The following abbreviations are used in this manuscript:

CK	Control
CF	Chemical fertilizer
CRA	Complex Conditioner A
CRB	Complex Conditioner B
CF+CM	Chemical fertilizer combined with cattle manure
CF+CRA	Chemical fertilizer combined with microbial inoculant Complex Conditioner A
CF+CRB	Chemical fertilizer combined with microbial inoculant Complex Conditioner B
H_2_SO_4_	Sulfuric acid
H_2_O_2_	Hydrogen peroxide
DM	Dry Matter
NAO	The nitrogen accumulation for an organ
NT	The nitrogen transport amount
NTR	The nitrogen transport rate
N-GCR	The nitrogen transport contribution to grain
PNA	The post-flowering nitrogen accumulation
NHI	The nitrogen harvest index
AEN	The nitrogen agronomic efficiency
NUE	The nitrogen use efficiency
ANR	The nitrogen fertilizer apparent recovery
APR	The phosphorus fertilizer apparent recovery
PFPN	The nitrogen fertilizer productivity
AN	Alkali-hydrolyzable nitrogen
AP	Available phosphorus
AK	Available potassium
PT	The phosphorus transport amount
PTR	The Phosphorus Translocation Rate
P-GCR	The phosphorus transport contribution to grain
PPA	The post-flowering phosphorus accumulation
PUE	The phosphorus use efficiency
PAE	The phosphorus agronomic efficiency
PFPP	The phosphorus fertilizer productivity
MZ_G_NA	Maize grain nitrogen accumulation
MZ_G_PA	Maize grain phosphorus accumulation
MZ_G_KA	Maize grain potassium accumulation
SB_G_NA	Soybean grain nitrogen accumulation
SB_G_PA	Soybean grain phosphorus accumulation
SB_G_KA	Soybean grain potassium accumulation
YLD	Yield
